# Prevalence and predictors of subclinical atrial fibrillation in hospitalized older adults

**DOI:** 10.18632/aging.203270

**Published:** 2021-07-01

**Authors:** Francesco Spannella, Federico Giulietti, Lorenzo Pimpini, Francesca Elena Lombardi, Serena Re, Paola Schiavi, Gina Dragano, Roberto Antonicelli, Riccardo Sarzani

**Affiliations:** 1 Internal Medicine and Geriatrics, IRCCS INRCA, Ancona, Italy; 2 Department of Clinical and Molecular Sciences, University “Politecnica delle Marche”, Ancona, Italy; 3 Cardiology Unit, IRCCS INRCA, Ancona, Italy

**Keywords:** atrial fibrillation, older, multimorbidity, hospitalized

## Abstract

Subclinical atrial fibrillation (SCAF) is associated with an increased risk of clinical AF, major cardiovascular events and death. Short-term evidence on SCAF in older populations is scarce, especially in the hospital setting. We performed a cross-sectional study on 60 multimorbid older consecutive patients (aged 80+) admitted to an Internal Medicine and Geriatrics Unit for acute medical diseases with no history of AF, in order to investigate prevalence and predictors of SCAF. Portable ECG monitoring was placed on admission and ECG recording lasted for 5 days. Mean age: 85.7±4.9 years. Female prevalence: 58.3%. High burden of comorbidities: 87.9%. All enrolled patients had CHA_2_DS_2_-VASc score ≥3. SCAF was detected in 16 patients (26.7%) and 11 patients (18.4%) had at least a SCAF episode lasting 6 minutes or longer. No clinical, laboratory and echocardiographic parameters predicted SCAF. Patients with ≥2004 supraventricular ectopic beats/24h (SVEBs/24h) had a 6-fold higher prevalence of SCAF than patients with <411 SVEBs/24h (p=0.038). Time to first SCAF episode was within 3 days of ECG recording in all enrolled patients. SCAF is highly prevalent in older adults hospitalized for acute diseases. This finding may affect clinical management and prognosis. Our study could foster larger multicenter studies in similar settings.

## INTRODUCTION

Atrial fibrillation (AF) is the most common arrhythmia in clinical practice. Its incidence increases dramatically with increasing age, also due to the aging-related cardiac remodeling [1]. Prevalence of AF in older adults is approximately 7.3% and subjects aged 80 years or older represent more than half of all cases of AF, with increasing estimates in coming decades [2]. However, the real prevalence of AF in the population, especially in older adults, is likely to be underestimated, as AF is often asymptomatic. Opportunistic pulse palpation or electrocardiogram (ECG) strips are most often unable to detect short AF episodes [3–5]. This is particularly true in high risk populations, such as patients with cryptogenic stroke, in which longer monitoring times showed to improve the detection rate of intermittent AF [4, 6]. Up to now, the latest European Society of Cardiology (ESC) guidelines on AF recommend opportunistic screening for AF by pulse palpation or ECG rhythm strip in patients ≥ 65 years and suggest more intensive ECG screening in individuals aged ≥ 75 years or those at high risk of stroke [7]. In hospital settings outside the intensive care units, continuous ECG monitoring and cardiac telemetry are not routinely used. However, several acute conditions could trigger supraventricular arrhythmias during hospitalization, even in patients with no history of AF. Furthermore, these events may be asymptomatic and therefore unrecognized. Older patients are often hospitalized for electrolyte disturbances, hypoxemia, acute organ failure and infections, and these abnormalities are associated to a greater risk of new-onset AF [8–10].

Subclinical AF (SCAF) is defined as AF episodes detected by implantable or wearable cardiac monitors and confirmed by visually reviewed intracardiac electrograms or ECG-recorded rhythm in subjects without symptoms attributable to AF, in whom clinical AF has not been previously detected [7]. Most of the data regarding SCAF derive from long-term studies on adult outpatients with known heart disease and implanted devices [11, 12] or continuous ECG monitoring in community-dwelling or selected populations (i.e. cryptogenic stroke) [4, 13, 14]. Conversely, short-term evidence on SCAF in older populations is scarce, especially in the hospital setting.

We performed an exploratory study to investigate both the prevalence and predictors of SCAF in multimorbid older adults hospitalized for acute medical diseases.

## RESULTS

### General characteristics

Sixty-five patients have been enrolled. However, five patients were excluded because they did not meet the minimum quality criteria for a satisfactory ECG recording (legible and artifact-free recording for at least 48 hours). Therefore, all the analyses were conducted on 60 patients. None of the enrolled patients had any symptoms attributable to AF episodes during hospitalization [15]. None of the enrolled patients died during ECG recording. Mean age was 85.7±4.9 years, with female prevalence (58.3%). General characteristics of the study population are summarized in Table 1. Anemia (Hbg < 12 g/dl) was found in 43 patients (71.7%) and eGFR < 60 ml/min/1.73mq was found in 27 patients (45.0%). No patients took oral anticoagulants. All enrolled patients had a CHA_2_DS_2_-VASc score ≥3. Echocardiographic characteristics of the study population are described in Table 2. More than half of the study population (57.1%) had left ventricular hypertrophy (LVH) and 56.8% had LV diastolic dysfunction. Most patients (82.5%) had a preserved left ventricular ejection fraction (LVEF ≥ 50%). The burden of co-morbidities was high in almost all of the subjects. The more prevalent co-morbidities were hypertension, heart failure (HF), diabetes mellitus, chronic obstructive pulmonary disease (COPD) and cognitive impairment.

**Table 1:** General characteristics of the entire study population and according to SCAF status.

**Clinical characteristics**	**Study population (n° 60)**	**Absence of SCAF (n° 44)**	**Presence of SCAF (n° 16)**	p*
Age (years)	85.7±4.9	85.5±4.2	86.3±6.5	0.671
Sex (females)	58.3%	61.3%	50.0%	0.430
BMI (kg/mq)	26.9±4.3	26.7±4.1	26.9±5.0	0.969
Arterial hypertension	83.1%	84.1%	80.0%	0.715
Type 2 Diabetes Mellitus	33.9%	34.1%	33.3%	0.957
History of TIA/Stroke	10.2%	11.4%	6.7%	0.603
History of CAD	11.9%	13.6%	6.7%	0.471
History of chronic HF	42.9%	52.3%	40.0%	0.412
History of COPD	33.9%	36.4%	26.7%	0.493
Cognitive impairment	34.5%	34.9%	33.3%	0.913
GIC (High comorbidity)	87.9%	90.7%	80.0%	0.273
ADL Hierarchy Scale: Assistance required	43.1%	44.2%	40.0%	0.854
ADL Hierarchy Scale: Dependence	41.4%	41.9%	40.0%	
Polypharmacy	77.6%	81.6%	63.6%	0.209
CHA_2_DS_2_-VASc score	4.6±1.2	4.8±1.2	4.1±1.2	0.060
Systolic BP (mmHg)	128.5±25.0	130.7±25.3	121.9±23.7	0.278
Diastolic BP (mmHg)	68.1±11.5	68.3±12.7	67.5±7.1	0.817
Heart rate (bpm)	80.0±14.5	78.5±13.1	83.8±17.7	0.232
Main admission diagnoses**				
Acute Decompensated HF	54.5%	61.0%	35.7%	0.101
COPD exacerbation	22.0%	22.7%	20.0%	0.826
Pneumonia	33.9%	31.8%	40.0%	0.563
Acute kidney injury	10.2%	13.6%	0.0%	0.131
Acute respiratory failure	55.9%	59.1%	46.7%	0.403
UTI or other infections	35.6%	40.9%	20.0%	0.144
*Admission lab parameters*				
NT-proBNP (pg/ml)	2082 (426-5160)	2366 (759-5136)	1075 (354-6904)	0.297
Hgb (g/dl)	11.0±1.6	10.9±1.6	11.3±1.7	0.425
WBC (n/mm^3^)	8595 (6235-11413)	8690 (6323-12682)	8290 (3343-10080)	0.292
eGFR (ml/min/1.73m^2^)	66.3±31.3	63.5±30.4	73.8±33.5	0.263
Serum sodium (mEq/l)	140.0±4.1	139.8±4.4	140.3±3.1	0.722
Serum potassium (mEq/l)	4.3±0.5	4.2±0.5	4.3±0.4	0.629
Albumin (g/dl)	3.3±0.5	3.2±0.5	3.6±0.5	0.387
Total cholesterol (mg/dl)	142.0±41.4	139.1±40.6	150.5±44.1	0.362
CRP (mg/dl)	8.0 (2.5-16.3)	8.0 (2.7-16.8)	6.7 (2.2-16.2)	0.525
Admission ABG parameters***				
pH	7.46±0.06	7.46±0.05	7.46±0.09	0.890
pO_2_ (mmHg)	58.5±15.1	56.6±14.4	64.3±16.2	0.128
pCO_2_ (mmHg)	42.0±13.0	42.2±14.2	41.3±8.6	0.809
P/F	253.9±63.7	247.3±59.0	269.5±76.7	0.300
HCO3^-^ (mmol/l)	28.5±6.6	28.4±7.2	28.9±5.0	0.813
Lactates (mmol/l)	1.4±0.7	1.5±0.8	1,3±0.4	0.391
*Cardiovascular therapy on admission*				
ACE-I/ARBs	51.0%	50.0%	54.5%	0.791
Diuretics	63.3%	63.2%	63.6%	0.977
Beta blockers	40.8%	44.7%	27.3%	0.299
Dihydropyridine calcium channel blockers	16.3%	18.4%	9.1%	0.461
Mineralocorticoid receptor antagonists	12.2%	13.2%	9.1%	0.717
Antiplatelet drugs	40.0%	43.2%	31.3%	0.404
Statins	20.4%	18.4%	27.3%	0.521

*Comparison between patients with SCAF and without SCAF.

**Each patient could have more than one admission diagnosis.

***Performed in 48 patients.

Continuous variables were expressed as mean ± standard deviation, or as median and interquartile range if markedly skewed. Categorical variables were expressed as percentage. Polypharmacy was defined as the use of 5 or more drugs.

SCAF: subclinical atrial fibrillation; BMI: body mass index; TIA: transient ischemic attack; CAD: coronary artery disease; HF: heart failure; COPD: chronic obstructive pulmonary disease; GIC: geriatric index of comorbidity; ADL: activities of daily living; BP: blood pressure; UTI: urinary tract infection; NT-proBNP: N-terminal pro–B-type natriuretic peptide; Hgb: haemoglobin; WBC: white blood cells; eGFR: estimated glomerular filtration rate; CRP: C reactive protein; ABG: arterial blood gas; ACE-I: angiotensin-converting enzyme inhibitors; ARBs: angiotensin II receptor blockers.

**Table 2:** Echocardiographic and continuous ECG monitoring parameters of the entire study population and according to SCAF status.

	**Study population (n° 60)**	**Absence of SCAF (n° 44)**	**Presence of SCAF (n° 16)**	p*
*Echocardiographic parameters*				
LVMI (g/mq)	99.4±32.2	96.0±30.5	115.2±39.0	0.233
LVEF (%)	55.8±9.5	54.9±9.3	58.4±10.1	0.323
LAVI (ml/mq)	36.4±11.2	36.4±11.3	36.6±11.4	0.977
E/E’	12.5±4.4	13.0±4.5	9.6±2.6	0.118
TAPSE (mm)	19.7±4.4	20.2±3.9	17.5±5.6	0.122
PAPs (mmHg)	32.8±7.9	33.0±8.0	32.0±8.4	0.799
*Continuous ECG monitoring parameters*				
Interatrial block (yes)**	36.7%	29.5%	56.3%	0.058
Mean HR (bpm)	72.6±12.2	71.0±11.7	77.1±12.9	0.087
N° VEBs/24h	1225 (278-2766)	764 (242-2355)	1996 (401-6002)	0.176
VEBs burden (%)	1.0 (0.0-3.0)	1.0 (0.0-2.0)	2.0 (1.0-5.5)	0.033
N° SVEBs/24h	724 (284-3941)	627 (249-2347)	2726 (443-6591)	0.032
SVEBs burden (%)	1.0 (0.0-3.8)	1.0 (0.0-2.8)	2.5 (1.0-5.8)	0.020
Presence of PSVT or VT	38.3%	18.8%	45.5%	0.060

*Comparison between patients with SCAF and without SCAF.

**Referred to ECG at admission.

Continuous variables were expressed as mean ± standard deviation, or as median and interquartile range if markedly skewed. Categorical variables were expressed as percentage.

SCAF: subclinical atrial fibrillation; ECG: electrocardiogram; LVMI: left ventricular mass index; LVEF: left ventricular ejection fraction; LAVI: left atrial volume index; TAPSE: tricuspid annular plane excursion; PAPs: systolic pulmonary artery pressure; HR: heart rate; VEBs: ventricular ectopic beats; SVEBs: supraventricular ectopic beats; PSVT: paroxysmal supraventricular tachycardia; VT: ventricular tachycardia.

### Prevalence and predictors of subclinical atrial fibrillation

**Figure 1:**
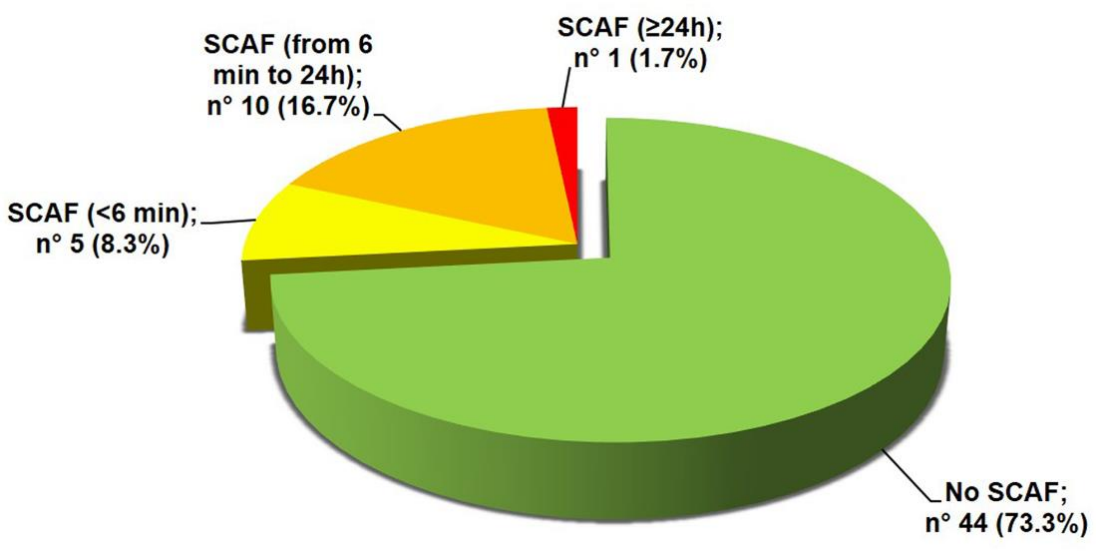
**Prevalence of SCAF.** SCAF: subclinical atrial fibrillation.

**Figure 2:**
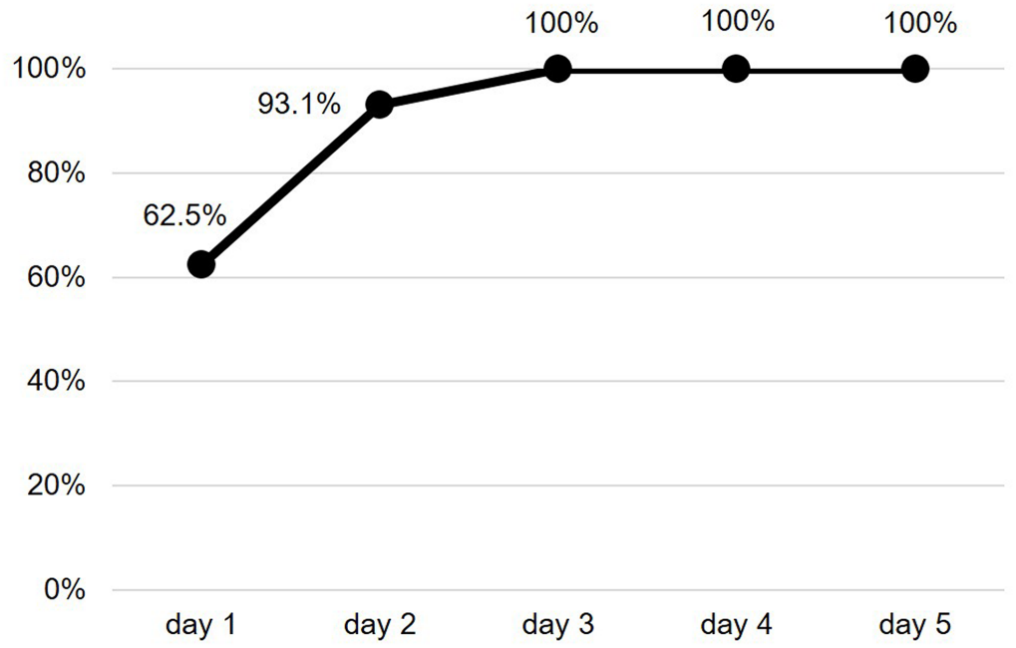
**Cumulative frequency of SCAF detection.** SCAF: subclinical atrial fibrillation.

The mean duration of the valid ECG recording was 4.2±1.4 days. A SCAF was detected in 16 patients (26.7%). The majority of these patients had at least a SCAF episode lasting 6 minutes or longer (Figure 1). Maximum heart rate during SCAF episodes was 183.3±45.4 bpm and the median duration of the episodes was 4.7 (1.7-10.3) minutes. The median of SCAF burden, defined by the percentage of analysable time spent in AF, was 3.7% (0.4-6.0%). No significant difference emerged between patients with or without SCAF regarding baseline clinical characteristics, admission diagnosis, laboratory and arterial blood gas (ABG) parameters or cardiovascular (CV) therapy on admission (Table 1). Moreover, both prevalence of anemia (72.7% vs 68.8%, p=0.762) and estimated glomerular filtration rate (eGFR) < 60 ml/min/1.73mq (50.0% vs 25.0%, p= 0.084) did not differ between patients with or without SCAF. The two groups showed similar echocardiographic parameters (Table 2), without differences regarding prevalence of LVH (57.7% vs 55.6%, p=0.911) and LV diastolic dysfunction (58.6% vs 50.0%, p=0.663). Patients with SCAF had almost double prevalence of interatrial block at baseline ECG compared to patients without SCAF, although without reaching statistical significance (Table 2). Patients with SCAF had both a higher daily number and burden of supraventricular ectopic beats (SVEBs) and a higher burden of ventricular ectopic beats (VEBs), as described in Table 2. After categorizing the number of SVEBs/24h into tertiles, patients with ≥ 2004 SVEBs/24h (3° tertile) had more than 6-fold higher prevalence of SCAF compared to patients with < 411 SVEBs/24h (1° tertile) [OR 6.2 (95% CI: 1.1-34.7), p=0.038]. Patients with SCAF had a higher mean duration of valid ECG recording, although not statistically significant (4.9±1.4 vs 4.1±1.4 days, p=0.067). Time to first SCAF episode was found within 3 days of ECG recording in all enrolled patients (Figure 2).

## DISCUSSION

In our study, we investigated both the prevalence and predictors of SCAF in multimorbid older adults with no history of AF and hospitalized for acute medical diseases. More than a quarter of study population had a SCAF episode during hospitalization, often lasting more than 6 minutes, but less than 24 hours.

### Prevalence of SCAF in clinical studies

Several previous studies investigated the prevalence of SCAF both in community-dwelling and selected high-risk populations, finding different results according to continuous or intermittent ECG recording, population screened, device used and duration of monitoring [3, 16]. A recent meta-analysis of studies, that performed a single time point screen for AF in the general population, found progressive increase in new AF detection rate with age, ranging from 0.34% (<60 years) to 2.73% (≥85 years) [17]. Studies on cardiac implanted electronic devices found high prevalence of SCAF, also because they often involved older subjects with significant structural heart disease, well-known risk factors for AF [18]. The ASSERT-II trial investigated the prevalence of SCAF ≥5 minutes, using subcutaneous electrocardiographic monitors, in older outpatients with risk factors, such as sleep apnea/obesity and left atrial enlargement, finding a detection rate of 34.4%/year [19]. In this trial, the median interval between the first episode of SCAF and clinical diagnosis of AF was only about 3 months [19], thus highlighting the propensity of these patients to later develop symptomatic disease. Lower rates were found using intermittent monitoring [20, 21]. A community-dwelling elderly population study (mean age: 79±5 years) found a prevalence of SCAF of 2.3% with 2 weeks of ambulatory ECG monitoring that increased to 4.1% with 4 weeks of monitoring [22]. Very recently, Gladstone DJ et al. detected a prevalence of 5.3% of asymptomatic AF lasting >5 minutes using a 2-week continuous wearable ECG patch monitor in individuals aged 75 years or older with hypertension and without known AF from primary care [23]. In the ASSERT-III, focused on older patients aged ≥80 years with hypertension and at least one additional AF risk factor who have been monitored continuously for 30–60 days, the authors found 15% of patients with at least one episode of SCAF ≥6 min [14]. The prevalence found in our study is likely the result of the peculiar setting in which our study took place. To the best of our knowledge, this is the first study that evaluated the prevalence of SCAF in such an older population (mean age: 85.7±4.9 years) in a hospital setting outside of intensive care units, using a continuous ECG recording.

### New-onset atrial fibrillation in the hospital setting

New-onset AF is defined as a new onset or a first detectable episode of AF, whether symptomatic or not, and not necessarily detected through screening [7]. Several acute conditions may be associated with new-onset AF during hospitalization. This could explain at least in part the high prevalence of SCAF found in our study. Previous studies found that patients admitted for both pneumonia and other infections, such as urinary tract or intra-abdominal infections, had a greater risk of new-onset AF during hospitalization [8]. New-onset AF risk is directly correlated with the severity of the infection and linked to both prolonged hospitalization and increased mortality risk [24, 25]. Pneumonia is often followed by CV complications, included AF. A recent report found a new-onset AF in 10.1% of patients admitted for pneumonia occurring early during admission, particularly associated with CHA_2_DS_2_-VASc score >3 and high burden of co-morbidities [26]. Overall, new-onset AF is identified in about 2% of all hospitalizations and the main risk factors include older age, comorbidity index, male sex, history of hypertension, myocardial infarction, HF, cerebrovascular disease, chronic lung disease and laboratory abnormalities (increased creatinine and decreased serum albumin level) [27]. The AF linked to acute conditions shows a high risk of recurrence and a worse long-term outcome [8, 27]. In addition to infections and the high burden of cardiorespiratory comorbidities, more than half of our older patients had cardiac or respiratory failure on admission. Acute systemic inflammation, cardiac overload, hypoxemia, neuro-hormonal activation, autonomic dysfunctions, typical of all these acute conditions, predispose to cardiac complications and arrhythmias [28–30].

### Possible clinical implications of SCAF

The clinical significance of SCAF is still uncertain. While SCAF episodes of shorter duration could be considered clinically irrelevant, previous studies found that SCAF episodes lasting more than 5-6 minutes are associated with an increased risk of clinical AF, ischemic stroke, CV events and mortality [11, 31–34]. Hence, the choice to identify and highlight those patients having a SCAF duration ≥ 6 minutes in our study, in agreement with previous studies [35], suggesting the possibility to follow these older patients more closely in order to prevent future adverse events. However, the risk of stroke in SCAF and the cost/benefit ratio of anticoagulation is still an open debate, given the lack of agreement in the published literature, especially in older people [36]. To date, there are no clear parameters of SCAF to initiate oral anticoagulation. In a post-hoc analysis of ASSERT study, only SCAF > 24h was associated with a significant increase in risk of stroke or thromboembolism [35]. However, a recent meta-analysis showed that it is not possible to define a SCAF duration threshold, because the relationship between SCAF duration and risk of stroke is likely to be continuous [37]. Overall, the risk of stroke would likely be lower than that of clinical AF [31]. It is important to note that shorter SCAF episodes are associated with higher probability of subsequent longer SCAF episodes and the progression to clinical AF over 2-year follow-up was found to be around 16% for detected SCAF lasting between 6 minutes and 24 hours [38, 39]. Moreover, patients with SCAF progression are also at greater risk for HF hospitalization [39]. Hence, a closer follow up of these older patients with short SCAF episodes could be advised. There is not always a temporal relationship between SCAF episodes and ischemic events, suggesting that SCAF, and in general AF, may be an epiphenomenon/marker of high risk patients, more than a direct risk factor [40]. At the same time, there are doubts regarding the real performance of CHA_2_DS_2_-VASc score in SCAF [18], although recent evidence showed a direct relationship between rate of stroke and CHA_2_DS_2_-VASc score also in SCAF [41]. Kaplan RM et al found that patients with a CHA_2_DS_2_-VASc score of 3 to 4 and an AF duration of >6 minutes had a risk of stroke >1%/year, an hypothetical threshold proposed for anticoagulant initiation in previous studies [41, 42]. It is important to note that all patients had a CHA_2_DS_2_-VASc score ≥3 in our study. Further studies may clarify the real benefits and indications of anticoagulation in older people with SCAF, especially regarding short SCAF episodes (≥ 6 minutes and <24 hours) [18, 43, 44]. The latest ESC Guidelines on AF suggest oral anticoagulation in selected patients at high risk of stroke (i.e. with previous stroke and/or age ≥75 years, or CHA_2_DS_2_-VASc ≥3) and a longer SCAF (≥5.5 hours), especially if high burden, mainly referring to SCAF detected by cardiac implantable electronic devices or cardiac monitors. However, a close follow-up for progression and risk factors control is also suggested for those with high risk of stroke and shorter SCAF episodes [7].

Besides a higher risk of stroke, HF hospitalization and mortality, older patients with AF have also a significantly lower cognitive performance than their counterparts in sinus rhythm, even after adjusting for age and the other CV risk factors [45, 46]. Both paroxysmal and persistent AF are associated with more than a 2-fold increased risk of silent cerebral vascular lesions [47] and there is an association between these findings in computed tomography or magnetic resonance in AF patients and risk of cognitive decline, even in the absence of manifest stroke [48]. Therefore, unrecognized AF, including SCAF, could contribute to accelerate cognitive decline in older subjects leading to disability and worse prognosis [49].

### Predictors of SCAF and ECG monitoring techniques

Previous population-based studies on outpatients found that increased LA volume and left atrial enlargement were associated with increased rate of SCAF [22, 50]. Furthermore, older age, male sex, hypertension, history of HF and higher CHA_2_DS_2_-VASc score were found to be independent predictors of SCAF [19, 33, 51]. In our study, we found no significant clinical or echocardiographic predictors of SCAF, likely due to the small sample analyzed, the very old age and the high prevalence of both CV risks and CV disease in our population. Interatrial block (IAB) is a conduction delay between the atria, detectable on the ECG. Its prevalence increases with age and has been found to be associated with an increased risk of AF [52]. In agreement with previous studies, we found higher prevalence of SCAF in patients with IAB in our study, although without reaching the statistical significance, likely due to the small sample size [53].

Frequent atrial premature beats (APBs) were found to predict paroxysmal AF during follow-up in patients with acute ischemic stroke and without known AF [54]. In EMBRACE trial, atrial premature beat count on 24h Holter ECG (APBs/24h) was the only significant predictor of SCAF, with a probability of AF that increased from 7-9% in patients with <100 APBs/24h to 40% in patients with ≥1500 APBs/24h [55]. Not by chance, the only predictor of SCAF in our study was the number of SVEB/24h. The identification of patients at higher risk of developing AF during hospitalization could help improving cardiac monitoring strategies and management.

In the outpatient setting, the longer the ECG monitoring, the higher the probability of finding AF episodes, although the optimal duration of continuous ECG monitoring in high risk populations is still unclear [56]. In our study, we found that SCAF episodes during hospitalization often had both a low burden and a short duration (median duration of about 5 minutes), suggesting that routine clinical examination (pulse palpation, auscultation and blood pressure measurement) or short-duration, standard 12-lead ECG are likely unable to detect SCAF in this setting. Interestingly, the first SCAF episode was within the first 3 days of ECG monitoring in the entire sample of our study. This finding could affect the duration of continuous ECG recording in future studies on this topic.

Screening campaigns in high-risk populations, such as subjects with previous stroke or outpatients aged more than 75 years, have been found cost-effective [57], although duration of screening, screening device, population to screen, and features of AF associated with significant stroke risk are still debated topics [3, 58]. The clinical significance and the correct approach/management of SCAF in older adults is still unclear, although it may be an opportunity to reduce both CV morbidity and mortality, by preventing new onset of stroke and hospitalizations [18].

### Study limits

The population taken into account (multimorbid hospitalized patients aged 80+ with no history of AF), almost never investigated in previous reports on this topic, represents the main novelty of our research. On the other hand, the small sample size due to this very particular population analyzed is the main limitation of our study. Future studies on larger samples could better clarify the prevalence and predictors of SCAF in older inpatients and, more importantly, could help understanding the implications of SCAF on clinical management and outcomes. Last, possible confounding risk factors occurred during hospitalization may not have been taken into account in the analyses.

## CONCLUSIONS

We found a high prevalence of SCAF in older adults hospitalized for acute medical diseases with no history of AF. Detecting a SCAF could significantly affect the clinical management of these subjects, in terms of both risk factors control and prevention of AF progression and complications. The latest ESC Guidelines on AF emphasize the role of a multidisciplinary approach with an integrated AF management team. In this context, geriatricians play a key role in managing older patients with AF and multiple morbidities. Our findings could foster larger multicenter studies on hospitalized older populations, in order to better clarify the clinical meaning and implications of this unrecognized condition, including anticoagulant therapy indications.

## MATERIALS AND METHODS

### Study design and population

We performed a cross-sectional study on older adults consecutively admitted for acute medical diseases, from January 2019 to July 2019, to the Internal Medicine and Geriatrics Unit of the Italian National Institute of Health and Science on Ageing (INRCA: Istituto Nazionale di Riposo e Cura per Anziani), which is the only organization specifically focused on geriatric care and gerontological research in Italy. Indeed, our hospital is dedicated to scientific research and care of older subjects (mostly aged 80 years or older), which are usually still excluded from clinical trials and in which scientific evidence is scarce. We took into account the following inclusion criteria: age ≥80 years, sinus rhythm on admission ECG, no history of AF reported by patient or by his medical records. We excluded patients having conditions with a life expectancy of less than 1 year (end-stage renal disease or dialysis, decompensated cirrhosis, advanced cancer, severe dementia or bed rest syndrome), decompensated hypo/hyperthyroidism, presence of implanted cardiac electronic devices (pacemaker or loop recorder), history of long QT syndrome or evidence of corrected QT interval (QTc) duration >500 milliseconds by the Fridericia formula. Patients admitted for acute diseases requiring continuous ECG monitoring (i.e. suspected cardiac syncope) were also excluded. All participants, or their legal representatives, gave their informed written consent and clinical investigations have been conducted according to the principles expressed in the Declaration of Helsinki. This study was approved by the local institutional ethics committee.

### Proceedings

A 12-lead ECG was performed in all patients on admission to evaluate the presence of sinus rhythm, according to our routine clinical practice. After taking into account the inclusion and exclusion criteria, a continuous ECG monitoring (LIFECARD CF, Spacelabs Healthcare Limited, Hertford, United Kingdom) was performed in all enrolled patients, within the first 12 hours from admission, for 5 days. The choice of 5 days was based on the mean length of stay in our ward found in previous studies [59]. This device allows a continuous ECG recording up to a maximum of 7 days using 3 electrodes and 3 channels. It notifies the patient after the displacement of an electrode allowing the correct replacement. Moreover, the correct positioning of the electrodes has been monitored 3 times a day by both the medical and nursing staff. At the end of the recording, data have been elaborated using a dedicated software and interpreted by a single expert cardiologist (LP) of the Cardiology Unit, in order to identify SCAF episodes. A valid diagnostic ECG monitoring needed at least 48 hours of readable recording. Minimum quality criteria for a satisfactory ECG recording were based on the 2017 ISHNE-HRS expert consensus statement on ambulatory ECG and external cardiac monitoring/telemetry [60]. During hospitalization, all patients were managed according to the usual “good clinical practice”, regardless of whether they have participated to the study or not.

### Clinical parameters

AF was defined by an irregular rhythm with absent P-waves lasting ≥ 30 seconds. Medical history and laboratory parameters were collected in each enrolled patient on admission. We took into account the following laboratory parameters: hemoglobin (Hgb), white blood cell count (WBC), creatinine, estimated glomerular filtration rate (eGFR), serum sodium and potassium, N-terminal pro-B-type natriuretic peptide (NT-proBNP), glycemia, C-reactive protein, albumin, total cholesterol. The eGFR was estimated using the CKD-EPI equation. Body mass index (BMI) was defined as the body mass divided by the square of the body height and was expressed in units of kg/m^2^. The CHA_2_DS_2_-VASc score was calculated according to the 2020 ESC Guidelines on AF [7]. The age-adjusted NT-proBNP cut-off of 1800 pg/mL, proposed by Januzzi et al, was used to diagnose acute decompensated heart failure (HF) [61]. An arterial blood gas (ABG) analysis was performed on admission as per clinical indication. During the hospitalization, a transthoracic echocardiographic evaluation was performed by the same physician, following a standardized protocol, to avoid inter-observer bias. The following main echocardiogram parameters were collected: left ventricular mass indexed to body surface area (g/m^2^) (LVMI), left ventricular ejection fraction (LVEF), left atrial volume index (LAVI), tricuspid annular plane excursion (TAPSE), systolic pulmonary artery pressure (PAPs), E/E’. Left ventricular hypertrophy (LVH) was defined as LV mass/body surface area (BSA) in mg/m^2^ > 115 (men) and > 95 (women) according to the 2018 ESC/ESH Guidelines [62, 63]. Left ventricular diastolic dysfunction was defined according to the 2016 ESC recommendations [64]. Regarding admission ECG, IAB was defined according to the following criteria: P-wave duration >120 msec without biphasic morphology in the inferior leads (II, III and aVF) for partial IAB, and P-wave duration >120 msec with biphasic morphology in the inferior leads for advanced IAB [53].

### Geriatric comprehensive assessment

As previously reported [65], to evaluate patients’ functional status, the 7-point MDS Activities of Daily Living (ADL) Hierarchy scale was used. The ADL Hierarchy scale groups activities of daily living according to the stage of the disablement process in which they occur [66]. The ADL Hierarchy Scale ranges from 0 (no dependence) to 6 (total dependence). ADL disability was categorized as follows: no impairment (ADL Hierarchy Scale score <2), assistance required (ADL Hierarchy Scale score 2-4), and dependence (ADL Hierarchy Scale score ≥5). Cognitive impairment was based on a previous documented diagnosis, given that the result of any cognitive test could have been altered by the acute phase. The Geriatric Index of Comorbidity (GIC) was used to determine the burden of comorbidities and it was categorized as low comorbidity (GIC classes 1 or 2) and high comorbidity (GIC classes 3 or 4) [67]. Polypharmacy was defined as the use of 5 or more drugs.

### Statistical analysis

Data were analyzed with the Statistical Package for Social Science version 13 (SPSS Inc. Chicago, Illinois, USA). A p-value less than 0.05 was defined as statistically significant. Continuous variables were checked for normality. Normal continuous variables were expressed as mean ± SD. Skewed variables were expressed as median and interquartile range. Categorical variables were expressed as percentages. The χ^2^ test was used to analyze the differences between categorical variables. The unpaired t test and Mann-Whitney test were used to compare quantitative variables.

Logistic regression analysis was used to evaluate the association between SCAF and number of SVEBs/24h categorized into tertiles.
